# Subchronic arsenism-induced oxidative stress and inflammation contribute to apoptosis through mitochondrial and death receptor dependent pathways in chicken immune organs

**DOI:** 10.18632/oncotarget.16960

**Published:** 2017-04-08

**Authors:** Hongjing Zhao, Ying He, Siwen Li, Xiao Sun, Yu Wang, Yizhi Shao, Zhijun Hou, Mingwei Xing

**Affiliations:** ^1^ Department of Physiology, College of Wildlife Resources, Northeast Forestry University, Harbin 150040, Heilongjiang, PR China

**Keywords:** arsenic, apoptosis, oxidative stress, inflammation, chicken

## Abstract

In many organ dysfunctions, arsenic and its compounds are well known to induce apoptosis by the mitochondria and death receptor apoptotic pathways in liver and airway. However, it is less reported that which signaling pathways contribute to excessive apoptosis of chicken immune organs, a major target of toxic metals biotransformation, which suffer from subchronic arsenism. In this study, we investigated whether the mitochondria or death receptor apoptotic pathways activated in the immune organs (spleen, thymus and bursa of Fabricius) of one-day-old male Hy-line chickens exposed to arsenic trioxide (As_2_O_3_), which were fed on diets supplemented with 0, 0.625, 1.25 and 2.5 mg/kg BW of As_2_O_3_ for 30, 60 and 90 days. We found that (1) Oxidative damage and inflammatory response were confirmed in the immune organs of chickens fed on As_2_O_3_ diet. (2) Subchronic arsenism induced typical apoptotic changes in ultrastructure. (3) TdT-mediated dUTP Nick-End Labeling (TUNEL) showed that the number of apoptotic cells significantly increased under subchronic arsenism. (4) As_2_O_3_-induced apoptosis of immune organs involved in mitochondrial pathway (decrease of B-cell lymphoma-2 (Bcl-2) and increase of protein 53 (p53), Bcl-2 Associated X Protein (Bax), caspase-9, caspase-3) and death receptor pathway (increase of factor associated suicide (Fas) and caspase-8). In conclusion, this work is the first to demonstrate that the activation of mitochondria and death receptor apoptosis pathways can lead to excessive apoptosis in immune organs of chickens, which suffer from subchronic arsenism, meanwhile, oxidative stress as well as subsequent inflammatory is a crucial driver of apoptosis.

## INTRODUCTION

In nature, arsenic is found among oxides and sulphur compounds, and mainly distributed through the environment by water cycling. Arsenic trioxide (As_2_O_3_) has recently been recognized as one of the most effective drugs for the treatment of acute promyelocytic leukemia. Every coin has two sides, a variety of studies indicated that inorganic arsenic and its methylated metabolites have paradoxical effects, namely, anticancer and carcinogenic effects [1]. Furthermore, epidemiological studies have shown that long term exposure to arsenic can increase the risk of cancers of lung, skin or bladder in man [2]. The Environmental Protection Agency and World Health Organization, in spite of that, have lowered the acceptable limit of arsenic in drinking water to 10 ppb, the concurrence of inorganic arsenic in groundwater has been reported in many Latin America and Asia countries, where inorganic arsenic concentration is up to 5300 ppb [3, 4]. For one thing, Gaworecki et al. [5] have reported that 25 ppm arsenic-exposed killifish during embryogenesis could initiate molecular changes that appeared to lead to aberrant muscle formation. For another in rats, As_2_O_3_ at a level of 5 mg/kg body weight (BW)/day elevated the levels of caspase-3 and nitric oxide and increased the expression of nucleic factor κB (NF-κB) p65 in the liver [6], which indicated the symptom of intoxication. Moreover, it has been also proved that longtime exposure to arsenic is deleterious to the liver [7], lung epithelial transformed cells [8], skin [9] and arsenic alters multiple cellular pathways, which include expression of cytokines, promotion and resistance of apoptosis and increasing oxidative stress [10], these alterations lead to disease manifestations. Recently, our studies have shown that subchronic exposure to As_2_O_3_ in excess of 0.625 mg/kg BW causes inflammation, oxidative stress and heat shock response in the liver [11], brain [12] and immune organs [13]. More and more serious conditions in arsenic-contaminated water and mining activities lead to the excessive arsenic accumulation through the food chains [14], human beings are exposed to arsenic species through their diets, therefore, they are susceptible to arsenic toxicity. Based on the abovementioned reports, arsenic and arsenic compounds toxic effects on health of animals and human beings, as well as their contamination in food and water have been a big problem in the environmental safety and public health.

Oxygen is the basis of creature life. However, metabolic imbalance and overproduced free radicals (including reactive oxygen species (ROS) and OH.) result in severe trauma and in contribution with several other environmental or genetic factors. As_2_O_3_ interacts with intracellular ROS causes cell damage [15], such as breaking the balance between oxidation and anti-oxidation, thus is responsible for not only for the increase of lipid peroxidation, but also for the reduction of antioxidant glutathione (GSH) levels, and the ability to resist OH. and inhibition of many anti-oxidative enzyme activities, such as glutathione peroxidase (GSH-Px), superoxide dismutase (SOD) and catalase (CAT) activities [16, 17]. In addition, ROS or oxidative damage has been implicated in the induction of apoptosis in the cecal tonsil of broilers exposed to nickel chloride [18]. Moreover, Yao et al. reported that cell apoptosis concurred with decreased antioxidant defense (GSH-Px activities) and increased lipid peroxidation (malondialdehyde (MDA) contents) in long-term selenium-deficient chicken muscles [19]. Another research in chicken liver suffering from aflatoxin B_1_ showed that oxidative stress triggered apoptosis via the activation of both mitochondrial and death receptor apoptotic pathways [20]. Further, arsenic (+3) can significantly increase ROS generation [21] and facilitate the major apoptotic signaling events: collapse of mitochondrial membrane potential, release of cytochrome c, down-regulation of anti-apoptotic protein Bcl-2 and subsequent activation of caspase-9 and caspase-3 [22]. In chicken hearts, subchronic arsenism-induced oxidative stress is also suspected to initiate inflammation, and ROS overproduction is thought to activate NF-κB pathway, which leads to increased expression of pro-inflammatory mediators such as tumor necrosis factor-α (TNF-α), prostaglandin E synthase (PTGEs), cyclooxygenase-2 (COX-2), inducible nitric oxide synthase (iNOS) [23]. In Caco-2 cells, chronic arsenic (+3) toxicity induces increases in the expression and release of the proinflammatory cytokines interleukin (IL)-6 and IL-8 [24], whose release depends on ROS production [25]. Lee et al. [26] demonstrated that iNOS is synthesized during inflammation and that iNOS supports nitric oxide production. Increased iNOS-mediated nitric oxide concentrations may lead to DNA damage and apoptosis. What's more, Yu et al. discovered that arsenic concentrations higher than 1 mM induced TNF-α release from mononuclear cells and caused apoptosis effect on T cells though TNF receptor I signaling pathway [27]. Overall, increased oxidative stress and subsequent inflammation may lead to increase in apoptosis [28].

In poultry, spleen, thymus and bursa of Fabricius (BF) are primary lymphoid organs responsible for the establishment and maintenance of the lymphocyte compartment, which is irreplaceable in maintaining organism in a favourable environment. Cui et al. reported that dietary NiCl_2_ in excess of 300 mg/kg impaired the innate and adaptive immunity in spleen, inhibited thymocyte and BF growth by arresting cell cycle, increasing apoptosis, and down-regulating cytokine expression levels [29–31]. A growing body of *in vitro* and *in vivo* evidence suggests that chicken immune organs are the target organ of heavy metals exposure, such as manganese [32], zinc [33] and so on.

Apoptosis, or programmed cell death, is an important way to maintain the cellular homeostasis between cell division and cell death. It is well known that apoptosis can be triggered via two principal signaling pathways: mitochondria apoptosis pathway and death receptor apoptosis pathway. However, there have been less reports focused on the apoptosis effects of As_2_O_3_ on immune organs in avian species at present. In this study, we demonstrated these two pathways could be triggered in the immune organs of chickens fed with As_2_O_3_ diet. Decreased antioxidant capacity and increased inflammation response suggested that oxidative stress and immune injury might be an important driver of excessive apoptosis of immune organs. Our research also found that the mitochondria and death receptor pathways were involved in As_2_O_3_-induced apoptosis of spleen and thymus, while only the former pathway was activated in BF cells, its exact mechanism needs further study. Overall, this study provides new and comprehensive evidences for further studying the effect mechanism of As_2_O_3_ on chicken.

## RESULTS

### Activities of antioxidant enzymes, GSH, OH. and MDA content in immune organs

**Figure 1:**
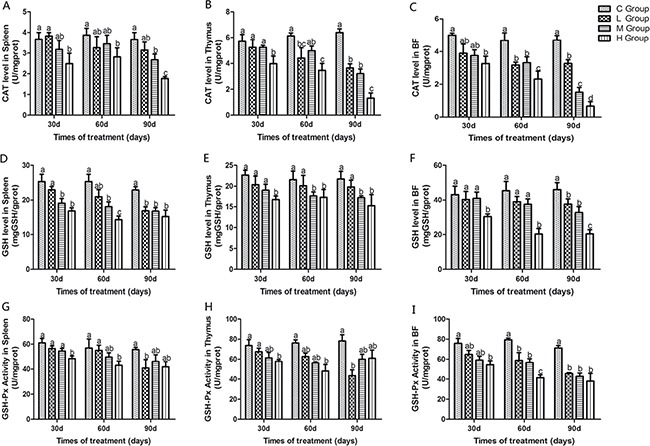
Effects of As_2_O_3_ on activities of CAT, GSH and GSH-Px at 30, 60 and 90 days Bars sharing a common letter are not significantly different (*P* > 0.05) in the same group. Each value represents the mean ± SD (*n* = 6).

**Figure 2:**
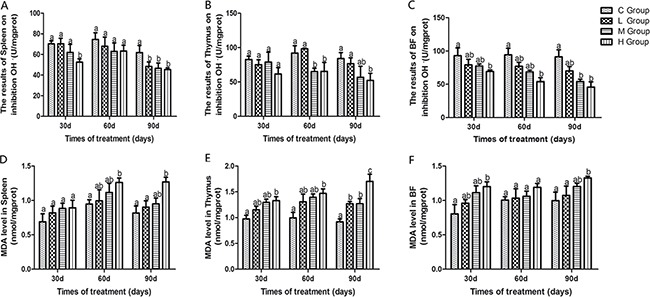
Effects of As_2_O_3_ on ability of inhibition OH and content of MDA at 30, 60 and 90 days Bars sharing a common letter are not significantly different (*P* > 0.05) in the same group. Each value represents the mean ± SD (*n* = 6).

Antioxidant enzyme activities, GSH, ability to resist OH. and MDA content in immune organs of chicken treated with As_2_O_3_ for 30, 60 and 90 days have been shown in Figures 1 and 2. Activities of CAT, GSH, GSH-Px, and ability to resist OH. in immune organs were decreased dose-dependently compared with the control groups at different time points. Their content decreased to the lowest levels in the high-dose groups compared with the control group (*P* < 0.05) except for the activity of GSH-Px in thymus on the 90th day, which the lowest level was appeared in the low-dose group. In contrast, MDA content in all of the As_2_O_3_-treated groups was higher than in the control group, correspondingly. And it reached its maximum value in high-dose groups.

### Histopathological analysis

**Figure 3:**
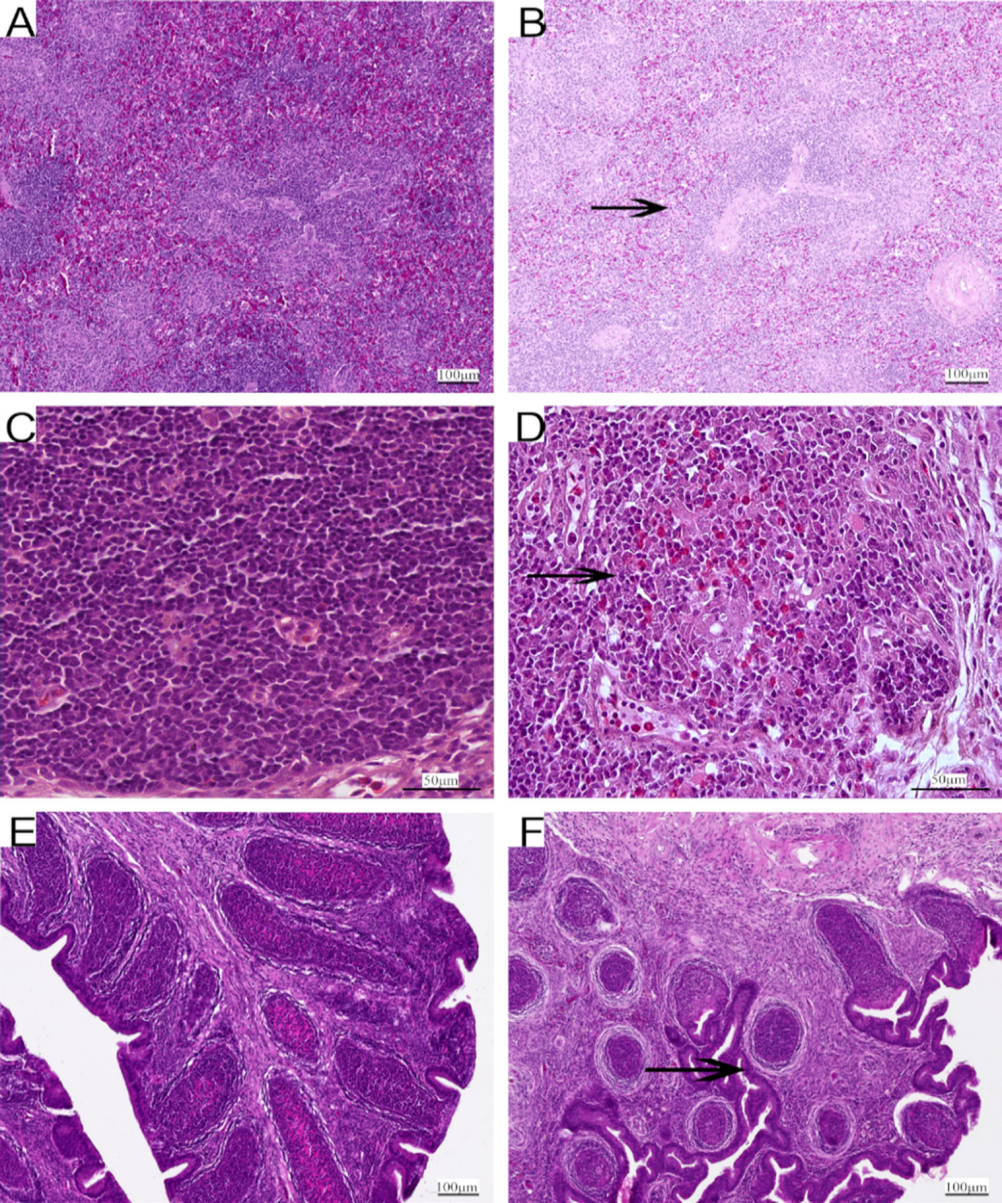
Representative photomicrographs of H&E-stained immune organs at 90 days Control group from spleen (**A**), thymus (**C**) and BF (**E**), high-As_2_O_3_ group from spleen (**B**), thymus (**D**) and BF (**F**) (×400) Black arrows stand for decreased lymphocytes, infiltration of heterophils in thymus and obvious atrophy of follicles in BF. Scale bars - 100 μm (a, b, e and f ), 50 μm (c and d).

As shown in Figure 3, some representative pictures on the 90th day illustrated the As_2_O_3_-induced histological changes in immune organs. The thymus, spleen and BF tissues samples from control groups showed normal histological structures in morphology in Figure 3A, 3C and 3E. Compared with that of control group, As_2_O_3_ treatment caused the number of white pulp lymphocytes of spleen decreased in high-dose group in Figure 3B, heterophils infiltration in thymus in high-dose group in Figure 3D. Follicles of BF were atrophic obviously in As_2_O_3_-treated group. At the same time, the boundary between cortex and medulla disappeared with a decreased number of lymphocytes, and connective tissue got significant hyperplasia in Figure 3F.

### Analysis of inflammatory mediators mRNA levels and iNOS protein level

**Figure 4:**
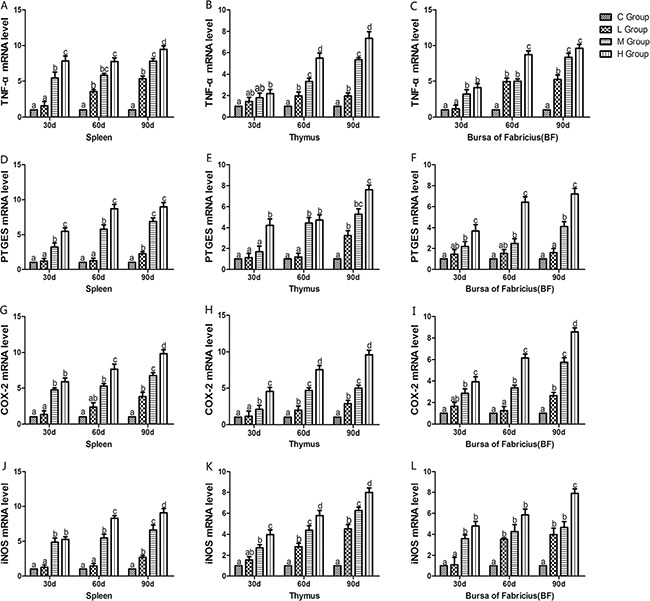
Effects of As_2_O_3_ on mRNA expression of TNF-α, PTGEs, COX-2, iNOS gene at 30, 60 and 90 days Bars sharing a common letter are not significantly different (*P* > 0.05) in the same group. Each value represents the mean ± SD (*n* = 6).

**Figure 5:**
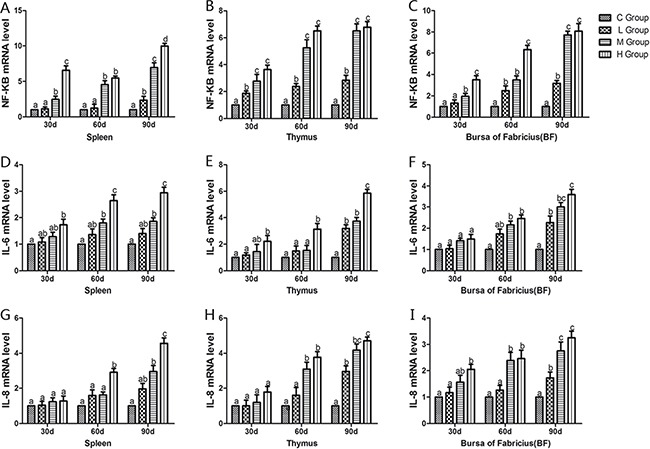
Effects of As_2_O_3_ on mRNA expression of NF-κB, IL-6 and IL-8 gene at 30, 60 and 90 days Bars sharing a common letter are not significantly different (*P* > 0.05) in the same group. Each value represents the mean ± SD (*n* = 6).

**Figure 6:**
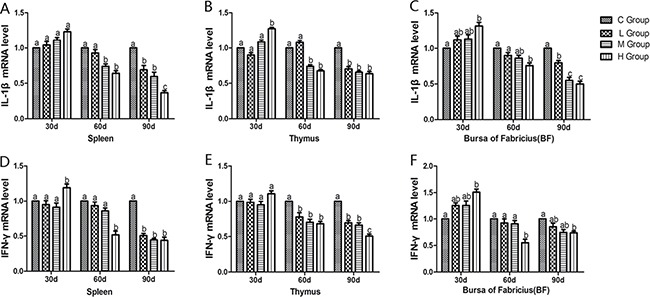
Effects of As_2_O_3_ on mRNA expression of IL-1β, IFN-γ gene at 30, 60 and 90 days Bars sharing a common letter are not significantly different (*P* > 0.05) in the same group. Each value represents the mean ± SD (*n* = 6).

**Figure 7:**
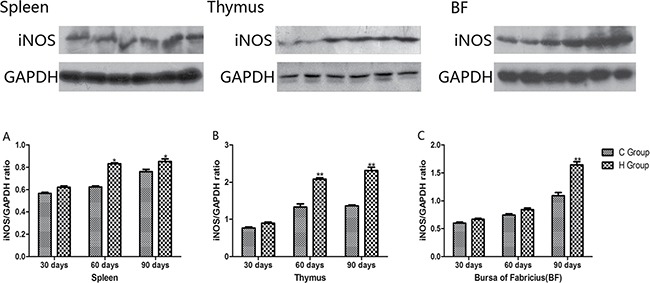
Effects of As_2_O_3_ on protein expression of iNOS at 30, 60 and 90 days *P < 0.05, compared with the control group Data are presented with the mean ± SD.

From the 30th to 90th days of age during the experiment, the mRNA levels of TNF-α, PTGEs, COX-2, iNOS, NF-κB, IL-6 and IL-8 were increased dose-dependently in treatment groups in three organs (Figures 4 and 5). Especially, levels in the medium and high-dose groups increased significantly compared with the corresponding control groups (*P* < 0.05) except the levels of IL-6 in BF as well as IL-8 in spleen and thymus on the 30th day, which showed no significant increase compared with the corresponding control groups (*P* > 0.05). On the 30th day, the transcriptional levels of IL-1β and IFN-γ in all these three immune organs reached their maximum values in the high-dose groups compared with the control groups (*P* < 0.05) (Figure 6). On the 60th and 90th days, they displayed decreases in a dose-dependent manner, and they reached the minimum values in the high-dose groups compared with the control groups (P < 0.05). Protein expression level of iNOS was consistent with its mRNA level treated by As_2_O_3_ which showed significant increase compared with corresponding control groups in three organs (*P* < 0.05) (Figure 7).

### Ultrastructural analysis

**Figure 8:**
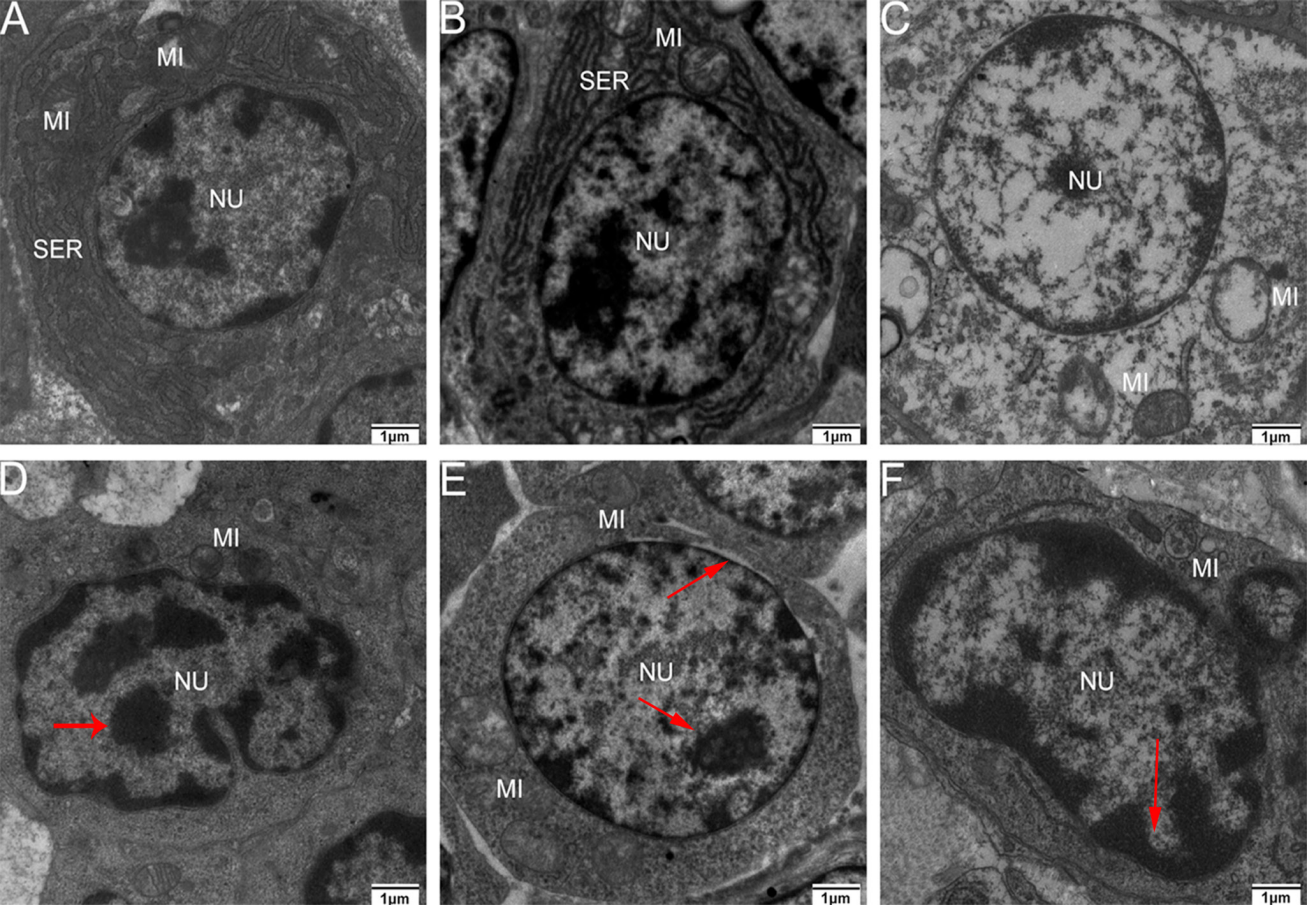
Transmission electron microscopy of immune organs at 90 days of age Control group from spleen (**A**), thymus (**B**) and BF (**C**), high-As_2_O_3_ group from spleen (**D**), thymus (**E**) and BF (**F**) (×2000) Red arrows stand for nucleus with lost structural integrity and chromatin condensation. Key: *MI*, mitochondria; *SER*, smooth endoplasmic reticulum; and *NU*, nucleus.

As shown in Figure 8, some representative pictures (immune organs samples on the 90th day of the experiment) illustrated the As_2_O_3_-induced ultrastructural changes in the immune organs. Electron microscopy revealed normal immune organs ultrastructures in control groups on the 90th day (Figure 8A, 8B and 8C). In contrast, As_2_O_3_ treatment caused extensive immune organs damage on the 90th day. A number of cells displayed morphological characteristic of apoptosis, including markedly swollen mitochondria with the degeneration or loss of cristae, blebbing of the membranes with cytoplasmic vacuolation, cell shrinkage, and chromatin condensation. Moreover, nucleus had lost its regular structure and was condensed while the nuclear membrane had appeared to lose structural integrity (Figure 8D, 8E and 8F).

### TdT-mediated dUTP Nick End Labeling (TUNEL) Assay

**Figure 9:**
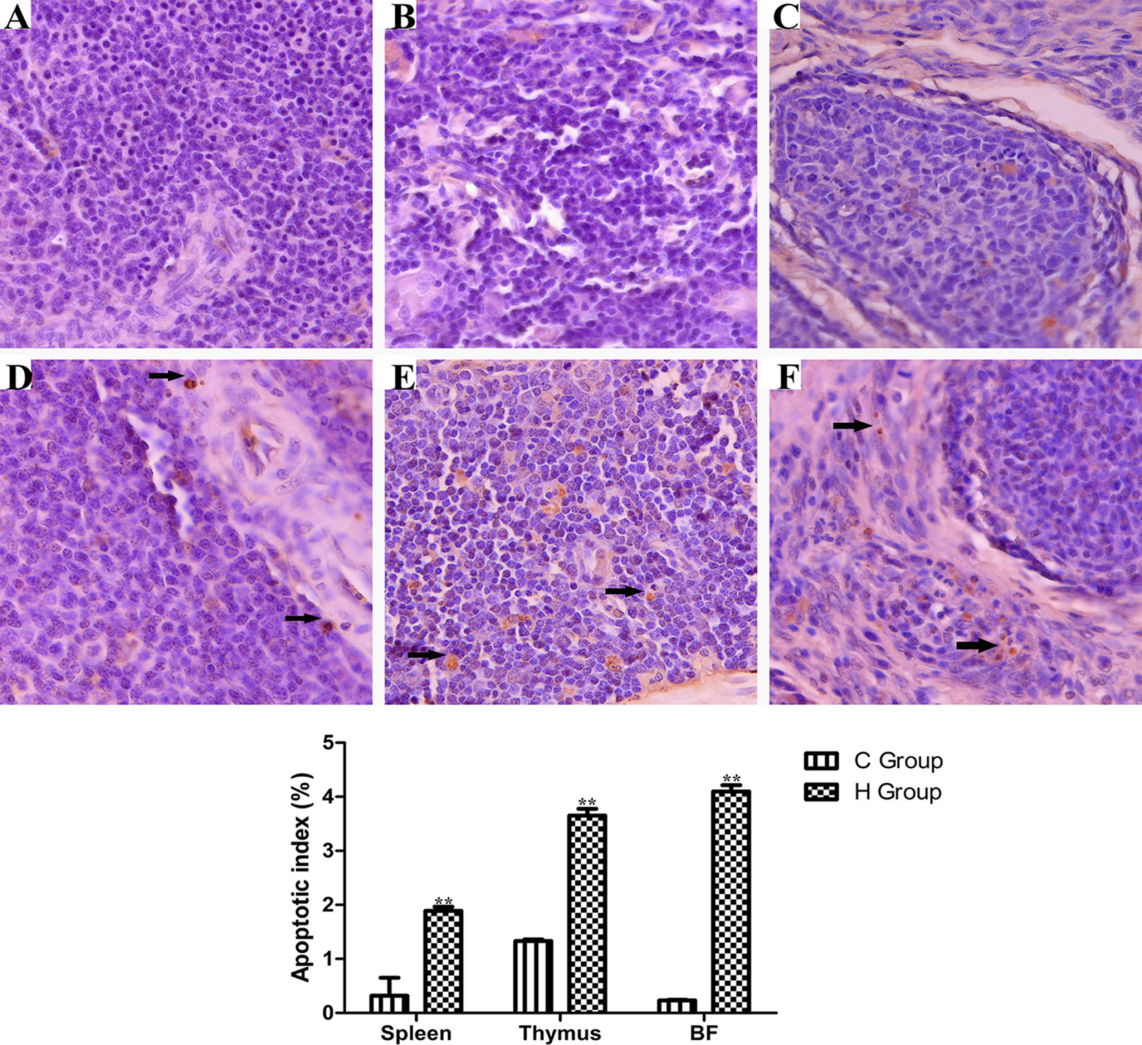
TUNEL staining (counterstained with hematoxylin, ×400) of dimmune organs at 90 days of age Control group from spleen (**A**), thymus (**B**) and BF (**C**), high-As_2_O_3_ group from spleen (**D**), thymus (**E**) and BF (**F**) **P < 0.01, compared with the control group. Data are presented with the mean ± SD (*n* = 6) Black arrows stand for apoptotic cells with brown-stained nuclei.

Effects of As_2_O_3_ treatment on apoptosis index in the immune organs have been presented in Figure 9. The number of apoptosis lymphocytes was significantly increased in high-dose groups compared with corresponding control groups (*P* < 0.05). Immune organs from each control group showed normal lymphocytes (Figure 8A, 8B and 8C), chicken treated with 2.5 mg/kg BW As_2_O_3_ in the diet for 90 days showed more apoptotic lymphocytes in the immune organs significantly (*P* < 0.05) (Figure 8D, 8E and 8F) compared with the control group.

### Determination of apoptosis-related genes’ mRNA levels

**Figure 10:**
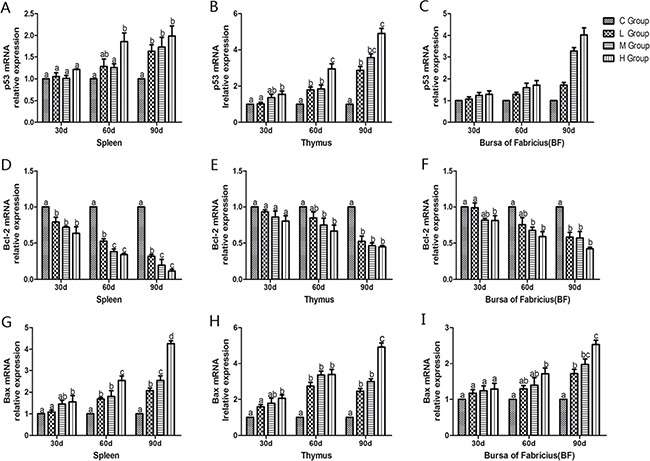
Effects of As_2_O_3_ on mRNA expression of p53, Bcl-2 and Bax gene at 30, 60 and 90 days Bars sharing a common letter are not significantly different (*P* > 0.05) in the same group. Each value represents the mean ± SD (*n* = 6).

**Figure 11:**
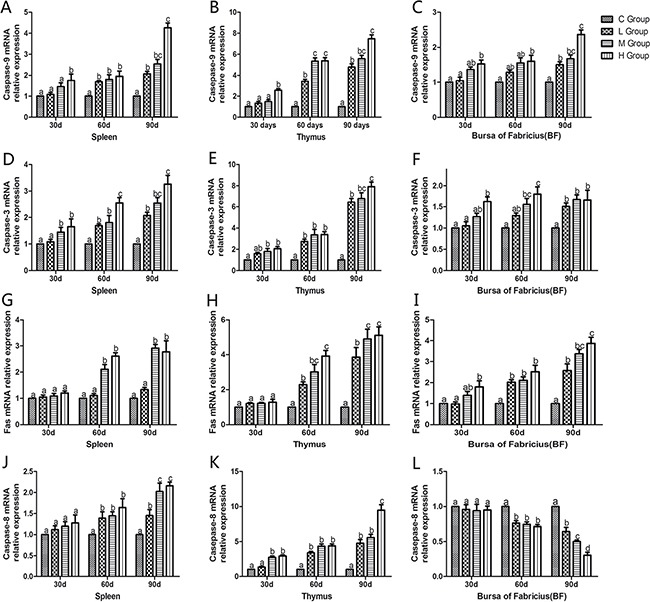
Effects of As_2_O_3_ on mRNA expression of caspase-9, caspase-3, Fas and caspase-8 gene at 30, 60 and 90 days Bars sharing a common letter are not significantly different (*P* > 0.05) in the same group. Each value represents the mean ± SD (*n* = 6).

To confirm the role of apoptosis in As_2_O_3_-induced injury, qRT-PCR was used to demonstrate change of mRNA levels of apoptosis-related genes in mitochondria apoptosis pathway (p53, Bcl-2, Bax, caspase-9, caspase-3) and death receptor apoptosis pathway (Fas and caspase-8) in As_2_O_3_-treated chickens (Figures 10 and 11). The mRNA levels of Bax, caspase-9 and caspase-3 in immune organs were increased dose-dependently on the 60th and 90th days and they increased to the maximum values in high-dose groups compared with the corresponding control groups (*P* < 0.05). However, the mRNA levels of p53 in spleen, BF and Fas in spleen and thymus, displayed no significant increases on the 30th days compared with the corresponding control groups (*P* > 0.05). The mRNA level of Bcl-2 in immune organs decreased at each As_2_O_3_ concentration and decreased to the lowest value in high-dose groups significantly compared with the corresponding control groups (*P* < 0.05). The mRNA level of caspase-8 in the BF was decreased dose-dependently, interestingly, it showed significant differences in the other two organs, which showed increased in a dose-dependent manner compared with the corresponding control groups (*P* < 0.05).

### Western blot analysis of apoptosis cytokines

**Figure 12:**
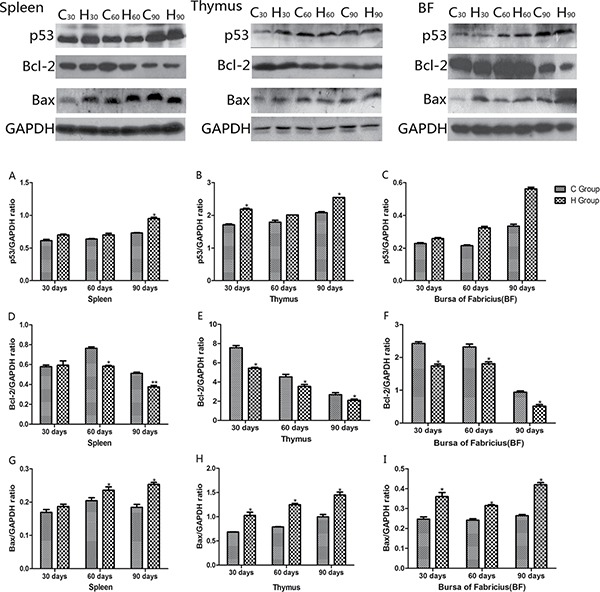
Effects of As_2_O_3_ on protein expression of p53, Bcl-2 and Bax at 30, 60 and 90 days *P < 0.05, compared with the control group Data are presented with the mean ± SD.

**Figure 13:**
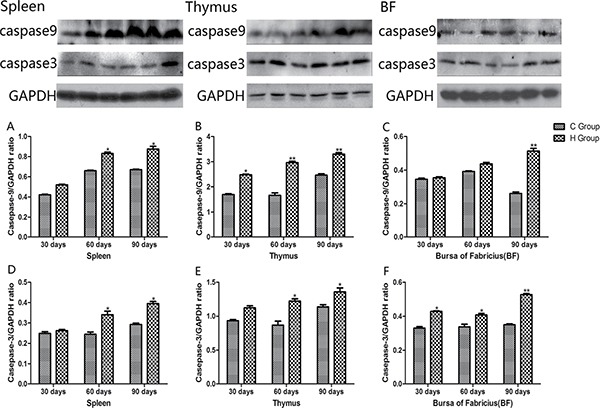
Effects of As_2_O_3_ on protein expression of caspase-9 and caspase-3 at 30, 60 and 90 days *P < 0.05, compared with the control group Data are presented with the mean ± SD.

Western blot analysis of apoptosis cytokines was shown in the Figures 12 and 13. We found that protein expression levels of p53, Bax, caspase-9 and caspase-3 increased significantly compared with corresponding control groups in spleen, thymus and BF (*P* < 0.05). Protein expression level of Bcl-2 was consistent with its mRNA level treated by As_2_O_3_ which showed significant decrease compared with corresponding control groups in three organs (*P* < 0.05).

## DISCUSSION

Arsenic is a nonmetal element, which widely distributes in water, food, drugs, and minerals that tends to cycle in our living environment [34]. As_2_O_3_ is quite toxic and it can induce lipid peroxidation, one of the main markers of oxidative stress, which leads to cytotoxic effect [35]. In the present study, decreased levels of antioxidants such as CAT, GSH-Px, and GSH, and the ability to resist OH. and an increased level of MDA were displayed in As_2_O_3_-treated chickens (Figures 1 and 2). The above results indicated that As_2_O_3_ exposure inhibited the antioxidant defense system, which led to oxidative damage and disturbances of metabolism and function in chicken immune organs. NO et al. [36] reported that airway oxidative inflammation may contribute to systemic inflammation through upregulation of Th17 immune responses in blood /liver and hepatic oxidative stress, namely, oxidative stress has a direct relationship with inflammation. Chickens are sensitive to As_2_O_3_, and a low dosage of As_2_O_3_ can induce immunosuppression, which showed the increases of NF-κB, TNF-α, IL-6 and IL-8 [11]. As revealed by histopathological analysis (Figure 3), an obvious atrophy of follicles in BF, infiltration of heterophils in thymus and decreased lymphocytes, were consistently observed in the As_2_O_3_-treated groups, which were in line with earlier researches [20, 37]. Results also displayed that the mRNA levels of TNF-α, PTGEs, COX-2, iNOS, NF-κB, IL-6 and IL-8 were increased in treatment groups in three organs (Figures 4 and 5), which were consistent with previous study [38]. The deleterious effects of As_2_O_3_ on anti-oxidative systems and immune defense have been suggested in chicken immune organs from above results.

Higher level of As_2_O_3_ depresses the activities of antioxidant enzymes, and then free radicals accumulate in the immune organs and induce the lipid peroxidation of the membrane. It is reasonable to propose that oxidative damage could occur in mitochondria and cause the release of proapoptotic proteins into the cytosol, which results in cellular apoptosis. Then, we tried to demonstrate apoptosis in the immune organs which occurred secondary to oxidative stress and inflammation, but it should be further clarified that if mitochondria or death receptor apoptotic pathways were both involved in this apoptotic procedure. Our study is the first to answer this question by using a chicken model suffering from subchronic arsenism.

In the present study, we performed an ultrastructure assay of chicken immune organs and found that As_2_O_3_ exposure to chicken caused typical features of apoptosis (Figure 7). In accordance with these definite morphological changes, apoptosis was further confirmed by TUNEL assay, which revealed the increased number of apoptotic cells in subchronic arsenism test (Figure 8). It indicated that As_2_O_3_ plays an important role in the induction of apoptosis in immune organs of chicken. Mitochondria pathway involves initial mitochondrial perturbation resulted from cellular stress or cytotoxicity [39]. Followed by the release of apoptogenic factors such as cytochrome c and apoptosis-inducing factor, the activation of initiator caspase-9 and effector caspase-3 can be also triggered by oxidative [40]. The Bcl-2 protein family, which locates mainly on the outer membrane of mitochondria, is the major regulators and effectors of the mitochondria pathway, it can be categorized into the anti-apoptotic proteins (such as Bcl-2-like proteins), and the pro-apoptotic proteins (such as Bax-like proteins) [41]. Moreover, p53 could participate in apoptosis through mitochondrial pathway by up-regulating the expression of Bax and down-regulating the expression of Bcl-2 [42]. In agreement with these theories, the increases in caspase-9, caspase-3, p53 and Bax as well as the decrease in Bcl-2 on protein contents and mRNA expression levels in our study suggested that apoptosis of immune organs was induced by As_2_O_3_ in 0.625 mg/kg BW and over, which by means of activating the mitochondrial pathway.

As a pro-apoptotic protein, caspase-3 is activated in the apoptotic cell both by mitochondria pathway and death receptor apoptotic pathway, and executes the apoptotic process [43]. Thus, we investigated the relative mRNA expressions of genes correlated with death receptor apoptotic pathway, such as Fas, which requires binding to the Fas ligand (FasL) [44]. Yang et al. [45] proved that compared with control, treatment with As_2_O_3_ at a level of 2 μM and 4 μM for 48 hours, resulted in increase of Fas gene expression by 28.31% and 56.74%, respectively. On the other hand, TNF-α can induce apoptosis through TNF-α receptor-mediated activation of Fas-associated death domain (FADD) protein, which brings about activation of caspase-8 [46]. Poulaki [47] reported that caspase-8 got methylation resulting in gene silencing which decreased expression of caspase-8, and prevented human retinoblastoma cells from apoptosis. In particular, the liberated NF-κB dimers enter nucleus, in which they regulate transcription of diverse genes to encode cytokines, growth factors, cell adhesion molecules, pro- and anti-apoptotic proteins [48]. In this experiment, mRNA levels of Fas, TNF-α, caspase-8 and NF-κB increased significantly compared with corresponding control groups in spleen and thymus on the 60th and 90th days. Our results demonstrated that the excessive apoptosis induced by As_2_O_3_ involved the death receptor pathway. Interestingly, mRNA levels of Fas displayed no significant increase in spleen (Figure 11G) and thymus (Figure 11H) compared with control groups on the 30th day (*P* > 0.05). Moreover, mRNA levels of caspase-8 in 0.625, 1.25 and 2.5 mg/kg BW groups showed lower values than that of control groups in the BF (Figure 11L), which indicted that death receptor apoptotic pathway wasn't activated until the 30th day in spleen and thymus. As for BF, this apoptotic pathway even hasn't been activated by As_2_O_3_ in present study. Our result is in line with previous study which showed the expressions of Fas, FasL, FADD and caspase-8 were no difference between the Aflatoxin B_1_ group and the control group, which evidenced that death receptor apoptotic pathway may not contribute to the excessive cell death of BF cells [41]. In summary, mitochondria apoptosis pathway plays a more extensive role than death receptor apoptotic pathway in chicken immune organs suffering from subchronic arsenism, and the latter pathway might be inhibited in BF cells, its exact mechanism needs further study.

Nevertheless, we also found that the mRNA levels of IL-1β and IFN-γ showed the highest value in H group on the 30th day and significant decrease on the 60th and 90th days following As_2_O_3_ exposure in immune organs compared with corresponding control groups (*P* < 0.05) (Figure 6). Lynch et al. suggested that Fas/FasL mediated activation of T lymphocyte apoptosis, controlled the inflammatory response to a certain extent, so as to avoid the occurrence of sustained, strong inflammatory response [49]. It was also consistent with Matzinger's theory that apoptosis cells do not stimulate the inflammatory response because of the lack of some risky signals [50]. Indeed, our results directly displayed that apoptosis cells do restrain the inflammatory response after subchronic arsenism, conversely.

**Figure 14:**
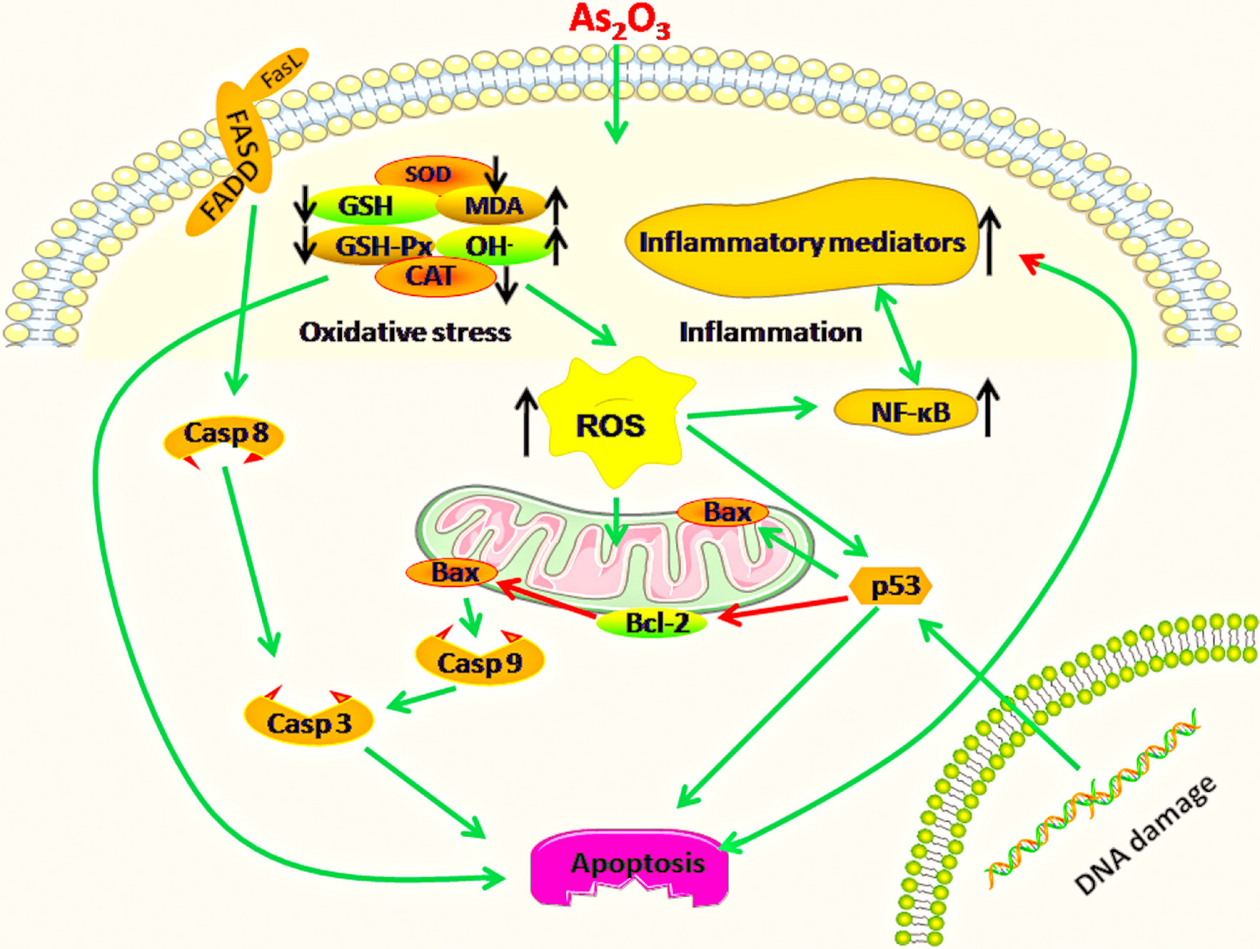
Diagram depicts the toxic effect of arsenic trioxide on chicken immune organs: Mitochondria and death receptor apoptosis pathways are activated because of excessive apoptosis in immune organs of chickens suffering from subchronic arsenism, and oxidative stress as well as subsequent inflammatory is a crucial driver during As_2_O_3_ exposure Green arrows mean promotion, red arrows mean inhibition. “↑”means up-regulation, “↓”means down-regulation.

In conclusion, the present study demonstrated that mitochondria and death receptor apoptosis pathways could be triggered in immune organs of chickens suffering from dietary As_2_O_3_ (Figure 14), meanwhile, oxidative stress and immune injury might be an important driver of excessive apoptosis in this study.

## MATERIALS AND METHODS

### Animals and treatment

A total of 72 one-day-old male Hy-line chickens were randomly divided into four groups (18 chickens per group). Chickens were housed in cages with electrical heaters, and provided with water by PVC pipeline and nipple drinkers as well as under-mentioned experimental diets ad libitum for 90 days. The composition of the diet was: Maize, grains 421 g/kg; wheat, grains 120 g/kg; full fat soy 180 g/kg; pea 100 g/kg; wheat bran 80 g/kg; limestone 80 g/kg; dicalcium phosphate 15 g/kg and sodium chloride 4 g/kg. This diet met the minimum requirements for energy and nutrients for chicken and without influencing results [51]. The highest dose of sub-chronic toxicity test can use 1/20 to 1/5 of the median lethal dose (LD_50_) [Supplementary Appendix 1], and median lethal dose of arsenic for chicken was 50 mg/kg BW [Supplementary Appendix 2]. To observe the dose-dependent dynamic change, we set the four groups of different dose levels: a control group (0mg/kg BW),a low As_2_O_3_-treated group (0.625mg/kg BW, corresponding 7.5 mg/kg feed), a middle As_2_O_3_-treated group (1.25mg/kg BW, corresponding 15 mg/kg feed), a high As_2_O_3_-treated group (2.5 mg/kg BW, corresponding 30 mg/kg feed). As_2_O_3_ was added into the food to make supplements according to the chicken median lethal dose of As_2_O_3_. Based on the mentioned method, we published some studies on arsenic exposure caused the changes of oxidative stress and heat shock in chicken [12, 13]. As_2_O_3_ was purchased from the New Technology Development Company, CHINA AGR UNIV. Animal studies, including animal care and all experimental procedures, were in accordance with the Animal Welfare Guidelines of Northeast Forestry University and the in-house guidelines of the Institutional Animal Care and Use Committee in Harbin, China. Animal experiment protocols were reviewed and approved by the Animal Care, Use and Ethics Committee at Northeast Forestry University (approval no. UT-31; 20 June 2014).

To observe the time-dependent dynamic change, we chose three time points (30, 60 and 90 days of age) for examining histopathological injury, immune, antioxidant and apoptosis parameter changes. On the 30th, 60th, and 90th days, six chickens in each group were selected randomly. Chickens were anesthetized and euthanized using injection of sodium pentobarbital (30 mg/kg BW), immune organs (spleen, thymus, and BF) were immediately excised, rinsed with ice-cold 0.9% NaCl solution. They were dried on filter paper and then marked.

### Determination of antioxidant enzyme activities and MDA levels

CAT, GSH, GSH-Px, ability to resist OH. and MDA levels in immune organs were determined in supernatants after isolated from chicken according to the method of the manufacturer's protocol of detection kits respectively (Nanjing Jiancheng Bioengineering Institute, Nanjing, China). Briefly, the activities of CAT, GSH, GSH-Px, ability to resist OH. and MDA level in immune organs were measured at 405 nm, 420 nm, 412 nm, 600 nm and 532 nm respectively.

### Histological observation

The immune organs were removed, fixed in 4% paraformaldehyde, dehydrated in ethanol and embedded in paraffin. Serial slices at 5 μm thickness were prepared and stained with haematoxylin and eosin (H&E), and examined by light microscopy.

### Quantitative real-time PCR

For the RNA quantification, immune organs (50 mg tissue; *n* = 6 /group.) were homogenized in liquid nitrogen with a mortar and pestle. According to the method described in the reference [13], total RNA was isolated from immune organs’ powders using RNAiso Plus reagent (Takara, China) according to the manufacturer's instructions. The concentration of RNA was measured by means of a spectrophotometer at OD_260/280_ ratio and then reverse-transcribed to cDNA using the PrimeScript RT Reagent Kit (Takara, China). Synthesised cDNA was diluted ten times with sterile water and stored at -80 °C before use.

Specific primers used for amplification were shown in Table 1. The relative mRNA levels of cytokines- and apoptosis-related genes were performed on a BIOER Line Gene 9600 Real-Time PCR System (Hangzhou, China) and determined with the FastStart Universal SYBR Green Master reagents (Roche, USA). The detailed conditions of PCR protocol and calculation method of each gene relative mRNA abundance are indicated in our previous research [13].

**Table 1:** A list of primers in qRT-PCR analysis of mRNA expression of the target genes

Genes	GenBank accession	Primer sequence (5′→3′)	Product size
NF-κB	NM205134	Forward: TCAACGCAGGACCTAAAGACAT Reverse: GCAGATAGCCAAGTTCAGGATG	162 bp
TNF-α	NM204267	Forward: GCCCTTCCTGTAACCAGATG Reverse: ACACGACAGCCAAGTCAACG	71 bp
PTGES	NM001194983	Forward: GTTCCTGTCATTCGCCTTCTAC Reverse: CGCATCCTCTGGGTTAGCA	115 bp
COX-2	NM001167718	Forward: TGTCCTTTCACTGCTTTCCAT Reverse: TTCCATTGCTGTGTTTGAGGT	84 bp
iNOS	NM204961	Forward: CCTGGAGGTCCTGGAAGAGT Reverse:CCTGGGTTTCAGAAGTGGC	82 bp
IL-6	NM204628	Forward:AAATCCCTCCTCGCCAATCT Reverse:CCCTCACGGTCTTCTCCATAAA	106 bp
IL-8	NM205498	Forward:GGCTTGCTAGGGGAAATGA Reverse:AGCTGACTCTGACTAGGAAACTGT	199 bp
IL-1β	NM204524	Forward: CAGCAGCCTCAGCGAAGAG Reverse:CTGTGGTGTGCTCAGAATCCA	86 bp
IFN-γ	GQ246226	Forward: GTGAAGAAGGTGAAAGATATCATGGA Reverse:GCTTTGCGCTGGATTCTCA	71 bp
p53	NM205264.1	Forward: GAGATGCTGAAGGAGATCAATGAG Reverse: GTGGTCAGTCCGAGCCTTTT	145 bp
Bcl-2	Z11961.1	Forward: ATCGTCGCCTTCTTCGAGTT Reverse: ATCCCATCCTCCGTTGTCCT	150 bp
Bax	XM001235092.3	Forward: GTGGTCAGTCCGAGCCTTTT Reverse: TCCATTCAGGTTCTCTTGACC	119 bp
Caspase-9	XM424580.5	Forward: ATTCCTTTCCAGGCTCCATC Reverse: CACTCACCTTGTCCCTCCAG	130 bp
Caspase-3	NM204725	Forward: CATCTGCATCCGTGCCTGA Reverse: CTCTCGGCTGTGGTGGTGAA	104 bp
Fas	XM421659	Forward: GCACTCGGTTTGGAGGTTGT Reverse: CGTGGCATTCCTGCTTCTT	197 bp
Caspase-8	NM204592	Forward: GGAAGCGGGAAGATATTGAG Reverse: GCCCAGGTAGGAAGCTAGAA	143 bp
GAPDH	K01458	Forward: AGAACATCATCCCAGCGT Reverse: AGCCTTCACTACCCTCTTG	182 bp

### Electron microscopy

The immune organs (size: 1.0 mm × 1.0 mm × 1.0 mm) were fixed immediately in 2.5% glutaraldehyde phosphate-buffered saline (v/v, pH 7.2), post-fixed in 1% osmium tetroxide (v/v) and stained with 4.8% uranyl acetate following dehydration. The samples were washed in propylene oxide and impregnated with epoxy resins. The semi-fine sections were contrasted with uranyl acetate and lead citrate for study via microscopy. The microphotographs were taken with a transmission electron microscope.

### In situ apoptosis detection

The samples were treated according to the manufacturer's protocol for the Colorimetric TUNEL Apoptosis Assay Kit (Takara, China). Quantitative evaluation of the apoptosis index was performed by manual counting of positively stained nucleus at 400 magnification. Apoptosis was determined by counting at least 100 cells from 5 to 6 sections of each immune tissue. Results were expressed as the percentage of TUNEL-positive cells among the total number of cells counted.

### Western blot analysis of apoptosis-related genes

For the protein quantification, a total of 50 mg tissue for each sample was rinsed in saline, cut and then homogenised in low intensity SDS Lysis Buffer (Beyotime, China). The homogenate was centrifuged, and supernatant was collected and determined using BCA protein assay kits (Thermo Scientific, USA).

For the western blot, the method was referred to our previous study [12]. Briefly, protein extracts (50 mg) were subjected to 12% SDS-polyacrylamide gels to separate target protein and then electrophoretically transferred to nitrocellulose membranes in Trisglycine buffer. Membranes were blocked with 5% skim milk at 37 °C, 50 rpm for 4 h, and incubated overnight with diluted primary antibody. To verify equal loading of samples, membranes were also incubated with GAPDH antibody (Beyotime, China). Then, membranes were incubated with horseradish peroxidase (HRP)-labeled Goat Anti-rabbit IgG (Beijing Biosynthesis Biotechnology, Co., LTD, China) at 37°C, 50 rpm for 1 h. Signals were detected by ECL western blotting detection kit (Thermo Scientific, USA). Target protein levels were normalized by GAPDH. The dilution (v/v) of primary chicken antibody: GAPDH, p53, Bcl-2, Bax, caspase-9 and caspase-3 were 1:1,000. Antibody against target protein was obtained from Wanleibio, China. And the dilution (v/v) of the secondary antibody was 1:7,500. The densitometry analysis of each blot was performed by employing Image J software, NIH, USA.

### Statistical analysis

Statistical analyses of all data were performed using SPSS for Windows software (version 13; SPSS Inc., Chicago, IL, USA). Differences between the mean values of normally distributed data were assessed with a one-way ANOVA (Dunnett's *t*-test) and two-tailed Student's *t*-test. *P*-values of 0.05 or less were considered to be statistically significant. Differences between means were assessed using Tukey's honestly significant difference test for post hoc multiple comparisons. All values were expressed as the means ± SD (standard error of means).

## SUPPLEMENTARY MATERIALS



## Abbreviations

As_2_O_3_: arsenic trioxide
BW: body weight
NF-κB: Nucleic factor κB
ROS: reactive oxygen species
GSH: glutathione
GSH-Px: glutathione peroxidase
MDA: malondialdehyde
SOD: superoxide dismutase
CAT: catalase
TNF-α: tumor necrosis factor-α
PTGEs: prostaglandin E synthase
COX-2: cyclooxygenase-2
iNOS: inducible nitric oxide synthase
IL: interleukin
BF: bursa of Fabricius
IFN-γ: interferon-γ
FasL: Fas ligand
FADD: Fas-associated death domain.
