# Supplementary material for: Subchronic arsenism-induced oxidative stress and inflammation contribute to apoptosis through mitochondrial and death receptor dependent pathways in chicken immune organs
Source: Oncotarget. 2017 Apr 8;8(25):40327–44. doi: 10.18632/oncotarget.16960 (PMC5522337; doi:10.18632/oncotarget.16960)
Supplement: Supplementary file 1 [file oncotarget-08-40327-s001.pdf]

Subchronic arsenism-induced oxidative stress and inflammation contribute to apoptosis through mitochondrial and death receptor dependent pathways in chicken immune organs

Supplementary Materials

Supplementary Appendix 1: The relationship between the highest dose of sub-chronic toxicity test and the median lethal dose (LD50)

环境毒理学基础

孟紫强 主编

高等教育出版社

化，每周调整饲料中受试物的浓度或灌胃体积，以维持恒定的剂量水平。

（三）染毒期限

染毒期限应根据受试物的种类和实验动物的物种而定，工业生产过程中接触的毒物的染毒期限相对可短一些，如亚慢性毒性试验染毒1－3个月，慢性毒性试验染毒6个月；环境毒物的染毒期限则相对要长一些，如亚慢性毒性试验染毒3－6个月，慢性毒性试验染毒一年以上；如果慢性毒性试验与致癌试验结合进行，则染毒期限最好接近或等于动物的预期寿命。有学者根据一些试验认为，大部分毒物在3个月的染毒期内即可出现毒性效应，延长染毒期不一定再出现新的毒性效应。最近，各种规范要求的染毒期限也在逐渐趋向于缩短。

为了探讨受试化学物对实验动物有无延迟毒性作用及引起的毒性变化是否具有可恢复性，可在染毒期结束后，各剂量组与对照组留部分动物继续饲养1－2个月，在此期间动物不再染毒，观察各项指标。

（四）剂量分组

在亚慢性和慢性毒性试验中，为了得到明确的剂量－反应关系，一般至少应设3个剂量组和1个阴性（溶剂）对照组。原则上高剂量组应能引起较为明显的毒性效应，但实验动物在染毒期间不应发生中毒性死亡，或死亡数少于动物数的10%；中剂量组应为引起轻微毒性的剂量，即观察到有害作用的最低剂量（LOAEL）；低剂量组的动物不应出现中毒反应，即相当于未观察到有害作用剂量（NOAEL）。具体选择剂量时，在亚慢性毒性试验中，可以参考急性毒性的有关参数，如以急性毒性的阈剂量作为亚慢性毒性试验的最高剂量，或以受试物 LD_{50} 的1/20－1/5为最高剂量；在慢性毒性试验中，可以选择亚慢性毒效应的NOAEL或其1/5－1/2为高剂量，以亚慢性毒效应的NOAEL的1/50－1/10作为慢性试验的中剂量组，1/100为低剂量组。虽然有上述原则可供参考，但在设计剂量时，必须根据受试化合物的特点，具体问题具体分析，必要时需要通过进行少量动物、较短时间的预试验来确定染毒剂量。高、中、低各剂量间要有适当的剂量组距，一般相差不小于2倍。

（五）观察指标

1. 中毒症状

在实验过程中，应每日观察实验动物有无中毒症状及程度，仔细观察动物的外观和行为有无异常。

2. 体重

动物体重可以反映受试物对实验动物的生长发育及一般状态的影响。实验过程中应每周称量动物体重一次。

3. 食物利用率

试验期间应注意观察并记录动物每日的饲料消耗量，并计算动物的食物利

In the case of a specific dose, in the subchronic toxicity test, acute toxicity parameters can be referenced, such as the threshold dose of acute toxicity as the highest dose of subchronic toxicity test, or to test 1/20~1/5 of the median lethal dose (LD_{50}) for the highest dose [1].

Supplementary Appendix 2: The provenance of LD50 of arsenic for chicken

东 北 农 业 大 学

学 位 论 文

三氧化二砷抗鸡 MD 肿瘤效应及其机理的研究

徐 世 文

指导教师姓名：石发庆 教授

申请学位级别：博士　　专业名称：临床兽医学

论文提交日期：2001 年 11 月　　论文答辩日期：2001 年 12 月

学位授予单位：东北农业大学　　学位授予日期：2001 年 12 月

答辩委员会主席：

评 阅 人：

二 00 一年十一月

3.2.2 SPF 雏鸡的 As_2O_3 急性毒性实验（LD_{50}）结果

3.2.2.1 临床症状与眼观剖检变化：

最急性于投药后 20 分钟出现临床症状，表现为呆立，少动，被毛松乱，流涎，摇头或甩头，挤堆，走路摇摆，站立不稳，后期有的出现兴奋，痉挛，最后倒地死亡。出现症状至死亡 15 分钟~7 小时不等。较慢的于投药后 1 小时出现中毒症状，表现基本同前。

剖检可见口腔粘液增多，嗉囊粘膜轻度充血，腺胃粘膜有出血斑，粘膜表面有多量粘液，腺胃乳头经挤压有多量粘液流出，肌胃角质层易剥落，角质层下有出血斑，十二指肠粘膜、小肠粘膜增厚，有斑状或弥漫性出血，盲肠、直肠无明显眼观变化。心冠脂肪和心尖脂肪胶样浸润，个别的有点状出血，心内膜有条纹状出血。肝肿大，有的表面有斑块状出血，胆囊充盈，胆汁色淡。肺充血，肾脏轻度肿大，有的脑膜有明显充血。其它组织器官无明显眼观变化。

3.2.2.2 急性 LD_{50} 测定结果

LD_{50}=62.6953mg/kg 体重，lgLD_{50}95%可信限为 47.0949~64.2430mg/kg 体重

45

3.2.2.2 Results of acute LD_{50} assay LD_{50} = 62.6953 mg/kg body weight, confidence limit of 95% lgLD_{50} is 47.0949~64.2430 mg/kg body weight [2].

REFERENCES

1. Ziqiang Meng. Basic environmental toxicology. Higher Education Press. 2003.

2 Shiwen Xu. The antagonistic effect and mechanism of arsenic trioxide on vMDV-infected chicken. vol. Northeast Agricultural University. 2001; 112.
